# EXPLORING CURRENT CHARACTERISTICS OF GEROTRANSCENDENCE: A QUALITATIVE STUDY OF OLD PERSONS LIVING IN TOKYO SUBURBS

**DOI:** 10.1093/geroni/igae098.2481

**Published:** 2024-12-31

**Authors:** Jun Kitayama, Hitoshi Igawa

**Affiliations:** Gakushuin University, Toshima-ku, Tokyo, Japan; Sakuragaoka Mental Health Clinic, Tama City, Tokyo, Japan

## Abstract

Tornstam (2005) proposed the concept of “gerotranscendence.” He identifies the phenomenon that the boundary between the self and the outer world is loosened in old age as gerotranscendence. The purpose of this study is to explore the experience of gerotranscendence among older people today living in the suburbs of central Tokyo. The participants, three men and five women who averaged 73.9 years old, attended a community association for older adults. The WHO-5-J scale was used to measure the participant’s mental health, and the overall score (21.4 out of 25 points) indicates that the eight participants were in good health. A semi-structured interview based on Tornstam’s gerotranscendence interview guide was conducted individually. The authors reviewed the collected narrative data and analyzed its individual and universal features. Unlike Tornstam’s idea, the results demonstrated that the participants are mainly living in actual life, in a preliminary state of gerotranscendence. The realization of psychological changes associated with aging were not often experienced. Most of them had not yet considered their views on death. They did not experience changes in their personality traits or personality integration. Interestingly, some participants mentioned that they reminisced about family members who had passed away or tried not to purchase new goods. It seemed that these usual expressions contained faint but significant indications of transformation in their views of life. This may suggest that clear phenomena of gerotranscendence seldom occur in healthy older adults. Therefore a better understanding of psychological transcendence may be achieved by focusing on individual small stories.

